# Supplementary material for: Age-specific income losses due to HPV-attributable cancers in Singapore
Source: PLoS One. 2026 Oct 30;21(10):e0361921. doi: 10.1371/journal.pone.0361921 (PMC13722152; doi:10.1371/journal.pone.0361921)
Supplement: Supplementary file 1 [file pone.0361921.s002.docx]

Authors response: We thank the editor for allowing us the chance to improve our manuscript, and reviewers for their considered feedback. We have included the point-by-point responses below.

**Reviewer 1**

Comment:

Respected Authors,

You have employed an interesting methodology to investigate this exceptionally important topic. The title of the paper is clear, and the abstract is well-structured and well-written. The introduction provides the necessary background information. The objective is clearly stated, and the methodology is described in detail, including all data sources. The results are clearly presented. In the discussion section, you have compared your findings with existing studies. The conclusion is consistent with the study’s objective and results.

Response: We thank the reviewer for looking over our paper and for their kind words.

**Reviewer 2**

Comment: Abstract
Line 19/20 are repetitive and don’t provide info on what kind of survival probabilities were used - are these cancer survival or general population?

Response: We have added some detail to the abstract regarding the use of Cox regression:

We applied sex- and cancer-specific Cox regressions to the Singapore Cancer Registry for annual predicted survival from 1992 to 2022. (Lines 19-20)

Comment: Line 22/23 what do these probability weights represent?

Response: This was unclear and has now been corrected:

Attributable fractions and vaccine effectiveness data for HPV 16/18 from the literature were used to estimate the effectiveness of bivalent HPV vaccination. (Lines 22-24)

Comment: Introduction

Line 45/46 the phrasing/punctuation here is off.

Response: This has been slightly amended for clarity:

In addition to mortality, HPV-related cancers can lead to significant disability, including loss of fertility and low self-esteem in cervical cancer survivors^4^ and facial disfigurement,^5^ trouble speaking and trouble swallowing^6^ in oropharyngeal cancer survivors. (Lines 47-50)

Comment (all below comments addressed in following response line):

Need to more clearly lay the ground work for what the existing vaccination strategy is in Singapore?

Is this bivalent vaccines?

What is the administration approach?

What ages are captured?

Line 50 - is this information specific to HPV-associated cancer? It ideally should be.

Line 50/57 - the connection between these two lines is not well made. There should also be more quantitative evidence presented that HPV-associated cancer rates amongst younger adults is increasing. We then end on “economic consequence of this possible shift is unknown” which seems to come out of nowhere.

Response: Upon reflection, we agree with the reviewer that the introduction did not provide a suitable background of Singapore’s strategy and the utility of HPV vaccination for our study. We have substantially edited the introductory section to incorporate much of the information from the discussion (as noted by the reviewer below), as well as providing greater detail around the current vaccine policy in Singapore across both females and males and our manuscript’s focus on HPV 16/18.

We also agree with the reviewer that the arguments around the age shift are less well-substantiated and distracting, and have removed them from the manuscript.

These changes now correspond to lines 51 to 82 in the updated manuscript. The discussion has also been amended to avoid duplication, and is further discussed below.

Comment: Line 59 - Need to better connect HPV vaccination to reduction in HPV cases and then connect this to cervical cancer elimination. There are other methods to controlling HPV spread that have been very important but these aren’t mentioned here?

Response: This context was initially missing from the manuscript and has now been added in several places:

Infection with high-risk HPV variants can lead to lesions, which may progress to cancer.^1^ (Lines 41-43)

Vaccination for HPV can prevent infection with the virus and is consequently crucial for cancer prevention.^8^ (Lines 51-52)

Comment: Line 65 - While there is gap in the literature that this paper fills, there is some existing work in this area and this should be mentioned (for comparison later). What about Hofmarcher (2018), Chesson et al. (2012), Bencina et al (2024)?

Response: We thank the reviewer for suggesting these works, which are indeed useful context for our paper. We have now added these papers to the discussion and explored how they relate to our paper’s findings.

Our results align with findings from Europe, where Bencina et al (2024) found that cervical cancer was the greatest contributor to productivity losses, but oropharyngeal cancer had the greatest losses per diagnosis. This is likely because both their study and ours utilised many of the same model parameters. Hofmarcher et al (2020) also evaluated indirect costs of cancer in Europe, noting that indirect costs comprised around a third of the total economic burden of cancer in high-income countries including the United Kingdom, Germany and France. Assuming a roughly comparable split of direct and indirect costs, extrapolating these results to Singapore suggests that HPV vaccination would be net cost-saving for cancer in both boys and girls.

In addition to savings from avoided income losses, HPV vaccination could be an effective measure to reduce the cost of cancer surveillance. Chesson et al (2012) investigated the potential savings from avoidable cervical cancer screening costs due to preventing HPV-associated disease. Approximately 82% of the direct costs of HPV were related to cervical cancer screening; policymakers may wish to consider that cancer-related costs are only a fraction of the overall economic burden of HPV that can be addressed by vaccination. (Lines 289-304)

Comment: Further evidence of the impact of HPV-related cancers in Singapore or the Asia Pacific region is needed.

Response: In re-designing the introduction, this point is now addressed in the context of cost-effectiveness. (Lines 53-71)

Comment: Methods

Line 81 needs to provide more detail around how the sex- and age-adjusted prevalence of each cancer in the Singaporean population.

Response: This is described in the *Annual cancer prevalence estimates* section. For clarity, we have added some detail:

To obtain smoothed estimates with standard errors, we fit a quasibinomial model for each cancer type and sex, with age at diagnosis as a predictor variable, and predicted the probability of diagnosis of each cancer at each age for both males and females. (Lines 135-138)

Comment: Line 143/144 unemployment data for nasopharyngeal is used as a proxy for oropharyngeal cancer.

Response: The reviewer is correct. We were not able to obtain oropharyngeal cancer rates and deemed nasopharyngeal cancer a suitable proxy.

In addition, upon reflection we opted to replace the generic male cancer employment rate with the employment rate for prostate cancer, as our previous estimates were based on overall cancer employment rate; we deemed prostate cancer a better proxy for anogenital cancers in men. This is now described in lines 152-157, but for the avoidance of doubt, we have added a new table, Table 1, to the manuscript which defines the simulation parameters.

Comment: Line 170 finally we have some information about the Singaporean government approach to HPV vaccine - this should come in the introduction and should be more informative.

Response: We agree with the reviewer that our introductory section needed to lay out this information in greater detail and have amended the introduction accordingly as per the previous comments.

Comment: Results

Tables need to be made more standalone - should be specified that this is SGD

Response: This has now been amended (Table 2)

Comment: Table 1 - Median lost income values have negative sign in front. A negative lost income would be an increase in income? This is inconsistent with how these values are presented in text.

Response: We thank the reviewer for raising this – the oversight has now been corrected.

Comment: I would like to see a lot more about sensitivity analysis, to test both structural and parametric assumptions used in the model. There are are some big assumptions made around attributable fractions and epidemiology and the potential impact of these assumptions on results should be explored.

Response: It is true that the model uses several assumptions that can be questioned. While probabilistic sensitivity analysis is designed to include the impact of this uncertainty on the final results, we agree that there were some parametric assumptions which may not have been properly substantiated.

In addition to clarifying the distributional assumptions by adding Table 1, we have also made some amendments to the model parameters by: changing the parameters used for attributable fraction and vaccine effectiveness to focus specifically on immunity from HPV 16/18; and incorporated the role of paid sick leave on return-to-work.

In terms of structural uncertainty, we agree that there was a particularly significant epidemiological phenomenon not covered, which was the role of herd immunity. In our original manuscript, we did not include herd immunity at all, but have now added it as a structural sensitivity analysis by estimating its impact on population-level HPV programmes. This now affects the overall estimation of program benefit at the national level.

Interestingly, we note that despite the changes to the parameters included here, there have not been major changes to our results, suggesting that our results were fairly robust to sampling uncertainty.

These changes are now included in both the methods, lines 197-208, and the results, throughout.

Comment: Discussion

Much of the papers outlined in the discussion should be brought up to the Introduction instead. The discussion should then be used to compare the results of the current paper to existing literature.

Response: We agree with the suggestion to reformat the paper. The introduction, as mentioned above, now frames the research in the context of the Singaporean health system and HPV vaccination programme. We have also, as suggested, redone the discussion as suggested, including some of the papers on global cancer costs and burden mentioned above.

We thank the reviewer for their helpful suggestions and believe the manuscript has been significantly improved as a result.

**Reviewer 3**

Comment: I found the paper is easy to read and impactful. I have few minor comments as follows:

1. I disagree with the authors' definition of productivity loss defined in the paper. As referred to Line 179-181, it only defined as difference between total expected income for healthy vs cancer diagnosed individual. Put it simply, it is loss of income, not productivity. I recommend the author to refer to standard productivity definition from International Labour Organization.

Response: We thank the reviewer for pointing out this issue. We have now aligned the language in the paper with the analysis to refer only to lost income, rather than productivity. We have also amended the title to avoid any misconceptions about the study’s scope and aims.

Comment: 2. I greatly appreciate if the authors can explicitly specify the model used in the study. I understand there is regression equation involved, but the technical specification is somehow missing from the paper.

Response: It is difficult to frame this model in the context of other, more traditional health economic models (e.g., Markov model, discrete-event simulation, etc). There is a Cox regression used to estimate mortality risks, but it is just one component of the calculations. As noted in our response to Reviewer 2, we have sought to clarify the methods, which together with Figure 1 and the model code, should clarify the modelling strategy applied.

Comment: 3. I also recommend that the authors incorporate relevant literature from leading economics and health economics journals to strengthen the paper’s academic positioning and enhance its visibility among economists who may be interested in pursuing similar research.

Response: This suggestion also aligns with the requests from Reviewer 2 to incorporate more references to the global burden of HPV and other vaccine-preventable cancers. We have now included a section in the discussion that seeks to align this work with the broader literature (Lines 289-304).

We thank the reviewer for their feedback and suggestions to improve our manuscript.
